# Research Progress of Hydromorphone in Clinical Application

**DOI:** 10.33549/physiolres.935354

**Published:** 2025-02-01

**Authors:** Li LIU, Meizi XU, Ji WANG, Yingxue HU, Zhiwen HUANG

**Affiliations:** 1 Department of Respiratory Medicine, Affiliated Renhe Hospital/Second Clinical Medical College of China Three Gorges University, Yichang, Hubei, China

**Keywords:** Hydromorphone, Pharmacological action, Route of administration, Cancer pain, Adverse effects

## Abstract

A common opioid analgesic for cancer pain and, to a lesser extent, non-cancer pain, is hydromorphone (HM). Oral formulations as well as subcutaneous, intravenous, and other routes are frequently used for its administration. Its pharmacokinetics and pharmacodynamics have also been extensively researched. This article examines the pharmacological properties of hydromorphone and the development of its use both domestically and internationally with the goal of serving as a reference for the sensible clinical use of this medication.

## Introduction

About 44.5 % of cancer patients report having pain, which is a typical symptom, especially in advanced cancer patients, where the frequency is greater (54.6 %). Additionally, the 30.6 % of patients will experience moderate to severe pain as the disease advances, which has a significant negative impact on the patients’ standard of living and quality of life and places a significant burden on families and society [1]. Currently, medication therapy is the mainstay of treatment for cancer pain. The preferred medication for treating moderate to severe cancer pain is an opioid analgesic, such as morphine. However, because of its side effects, which include respiratory depression, more novel opioids are being researched and developed. A number of studies have demonstrated that hydromorphone, a semi-synthetic derivative of morphine, has more severe side effects and unstable pharmacokinetics [2]. There have been numerous clinical investigations, and hydromorphone has recently made a breakthrough not only in cancer pain, but also in non-cancer pain. Oral, intravenous, subcutaneous, and intrathecal administration are common routes of administration for hydromorphone. These routes can be paired with mechanical or electronic pump technologies, and each has its own set of benefits and drawbacks. As a result, staying up to date on the most recent developments in the therapeutic use of hydromorphone may enable more thorough, focused, and customized care.

## Clinical pharmacology of hydromorphone

Due to the hydrogenation of the double bond between positions 7 and 8 and the oxidation of the hydroxyl group at position 6 to a ketone, hydromorphone, an opioid analgesic and semi-synthetic derivative of morphine, has a 5–10-fold greater analgesic effect than morphine and crosses the blood-brain barrier more easily [3]. There are many ways to give hydromorphone, including orally, parenterally (intravenously, subcutaneously, and intramuscularly), and intrathecally. Its primary targets are μ opioid receptors, and it also has a weak effect on δ receptors, but not on κ and ɛ receptors. It is metabolized by the liver and eliminated by the kidneys. Hydromorphone is converted to hydromorphone 3-glucuronide (H3G) and dihydromorphine 3-glucuronide by hepatic glucuronide without being metabolized by the cytochrome P450 (CYP) system, avoiding interactions with drugs metabolized by CYP and producing no metabolites of morphine: morphine-3-glucuronide (M3G) and morphine-6-glucuronide (M6G), thus reducing the incidence of renal impairment and adverse effects such as respiratory depression [2]. H3G and M3G, however, have been shown in studies to be able to produce neuroexcitatory effects; as a result, in clinical practice, a drug shift or drug dose adjustment should be taken into consideration if patients experience other neurotoxic reactions like tonic seizures [2]. In terms of pharmacodynamics, the analgesic effect of hydromorphone is 5–10 times higher than the equivalent dose of morphine, with significant individual variability depending on the patient, and the onset of action is relatively faster, about 5 min for intravenous administration; about 62 % of hydromorphone administered orally is eliminated by the first pass through the liver, and the elimination half-life through the blood-brain barrier is 28 min, compared with 166 min for morphine, which is much longer than that of hydromorphone.[Fig f1-pr74_41]

## Application of various hydromorphone routes of administration for cancer pain

### Oral

Oral delivery of hydromorphone is the quickest and least expensive method of administration. The oral dosage forms of hydromorphone include powder, solution, capsule, immediate-release tablets, and modified-release tablets. Hydromorphone is taken by mouth as a capsule. There are two types of capsule available: quick-acting capsules (Palladone®) and modified-release capsules (Palladone® SR). However, in 2005, the drug was withdrawn from the market due to the increase in blood levels after taking it with alcohol [4], and in recent years, there have been new guidelines pointing out the safety of Palladone®, but there is still a lack of relevant clinical trials. The oral immediate-release form has an onset of action of about 30 min and a duration of action of about 4 h. Meanwhile, newer formulations, such as the push-pull pump and other modified extended-release oral forms, including the osmotic release oral system (OROS), provide a prolonged effect, lasting 12 to 24 h depending on the specific formulation [5]. Notably, oral hydromorphone extended-release tablets are less effective in treating cancer pain due to spinal metastasis with numbness than tapentadol and methadone [6].

### Subcutaneous

Because of its great solubility and compatibility with other medications, hydromorphone is especially well suited for subcutaneous injection. Using subcutaneous infusion of opioids reduces pain, nerve damage risk, and infection risk [7]. The initial dose of hydromorphone given subcutaneously ranges from 0.5 to 1.0 mg every hour, which can be adjusted according to the severity of pain the patient is experiencing. For patients already using other opioids, such as morphine or fentanyl, their dosages can be converted to the hydromorphone equivalent, with the conversion ratio of oral to subcutaneous administration being approximately 2:1. Continuous subcutaneous injection (CSCI) and patient-controlled analgesia (PCA) are the two commonly used subcutaneous injection methods [8]. Twenty-five patients with advanced tumors received hydromorphone *via* self-controlled injection and continuous subcutaneous infusion; the two administration methods were switched after 3 days and had similar analgesic and side effect profiles [8]. Additionally, studies have shown that patients with severe opioid use disorder who are already receiving subcutaneous opioid injections may not respond effectively to oral opioid agonist medications for various reasons. However, subcutaneous or intravenous administration of opioids has become an established treatment option for patients who have failed conventional oral or opioid therapies, often resulting in favorable therapeutic outcomes [9].

### Intravenous

The most notable characteristics of intravenous administration are a quick onset of action, low first-pass elimination, and high bioavailability. Upon intravenous administration, the drug immediately enters the bloodstream, providing quick relief. However, maintaining intravenous access can be financially burdensome for patients, requires patient compliance, and depends on the patient’s overall health and nutritional status [10]. In cases where rapid dose adjustment is needed, or for managing severe pain, subcutaneous administration is preferred. Within five minutes of intravenous injection, the analgesic effect begins, and the maximal analgesic effect is felt eight to twenty minutes after the maximum plasma concentration [2]. The equivalent dose when moving from oral to intravenous administration is approximately 3:1 to 2:1. The initial dose is 0.1–0.2 mg when administered *via* PCA or intravenous infusion [5]. When considering special populations, such as cancer patients with renal impairment, studies have shown that systemic clearance of hydromorphone is reduced by 52 % to 69 % compared to patients with normal renal function [11]. This reduction in clearance underscores the importance of adjusting dosing for patients with renal impairment to ensure safe and effective pain management. Such insights are crucial for clinicians managing cancer pain with intravenous hydromorphone.

### Intrathecal

If patients do not benefit from other routes of administration, intrathecal injection may be considered an effective therapy for advanced recalcitrant cancer pain. It offers a prolonged duration of action, has few systemic side effects, but requires specialized technical expertise [12]. In patients with advanced cancer pain, self-administered intrathecal hydromorphone analgesia can substantially lower patients’ visual analogue scale (VAS) scores and increase satisfaction [13]. Additionally, in a comparative study of patients with intractable cancer pain, intrathecal hydromorphone was associated with fewer side effects compared to intrathecal morphine [14]. One of the practical advantages of intrathecal hydromorphone is its stability; hydromorphone administered intrathecally can be stored for up to 15 days at 25 °C and 37 °C while maintaining over 94 % of its initial concentration. This allows for biweekly replacement of the drug reservoir without compromising the effectiveness of pain relief [15].

### Patient-controlled analgesia

Patient-controlled analgesia (PCA) is a pain management technique in which healthcare professionals employ self-operating analgesic devices to pre-set analgesic medication dosages based on the patient’s condition and level of discomfort, and then hand over the patient to realize pain “self-management”. PCA technology has been extensively utilized for postoperative analgesia since the 1970s. Recent years have seen a growing promotion of PCA for the treatment of cancer pain, primarily in the areas of rapid opioid titration in patients with severe cancer pain, maintenance treatment of refractory cancer pain, and control of breakthrough pain. This is due to the technique’s successful application in the field of postoperative analgesia. The application of computer-based PCA in clinical settings enables precise control of drug delivery rate via microcomputer technology. This enables flexible adjustment of drug delivery parameters based on patient condition and makes the device more suitable for treating cancer pain. According to the various drug administration channels, PCA electronic pumps can be integrated with other drug delivery techniques. These include intrathecal PCA, regional block PCA, patient-controlled intravenous analgesia (PCIA), patient-controlled subcutaneous analgesia (PCSA), and so forth. The most widely used PCA administration routes for treating cancer pain are PCIA and PCSA. However, there are some differences between the benefits and drawbacks of using PCIA and PCSA, and since hydromorphone has been introduced relatively late for cancer pain management, the dosage recommendation for treating cancer pain using PCIA/PCSA techniques are not standardized. Nevertheless, this issue deserves more attention [16]. In a case of advanced cancer patients with refractory pain, daily acupuncture point treatment during HM pump analgesia improved pain control, reduced adverse reactions, and enhanced the quality of life. This suggests that HM pump analgesia can be effectively combined with traditional Chinese medicine Treatment [17].

## Application of hydromorphone for non-cancer pain

### Acute pain

Acute pain is defined by the International Association for the Study of Pain (IASP) as pain that is new and of short duration. It typically arises from surgical trauma, tissue injury, or certain medical conditions, and is one of the most common complaints in clinical practice [18]. Hydromorphone has been shown to reduce the intensity of transient pain by approximately 50 %, significantly alleviating patient discomfort. Additionally, radiofrequency catheter ablation for transient pain during atrial fibrillation surgery demonstrated that hydromorphone is more effective than fentanyl [19]. In cases of acute severe pain, intravenous hydromorphone at a dosage of 0.015 mg/kg was found to be more effective than morphine at 0.1 mg/kg, with fewer adverse effects [2]. The potency ratio of epidural morphine to hydromorphone for pain relief after acute hemorrhoidectomy was established at 3:1 [20]. These findings highlight the significant efficacy of hydromorphone in acute pain management, offering effective pain relief with fewer side effects, which supports its further clinical application.

### Chronic pain

Chronic pain is defined by IASP as pain that lasts longer than the expected duration of treatment for a given disease or injury [18]. Typically, chronic pain lasts for at least three months and is influenced by a complex interplay of biological, psychological, and social factors. Richarz *et al*. [21] found that more patients preferred hydromorphone due to its convenience, resulting in fewer treatment discontinuations due to adverse effects. This enhanced adherence to treatment also lowered the risk of relapse after discontinuation. Most patients with chronic pain can successfully transition to alternative therapies within 2–4 weeks, with approximately 80 % achieving satisfactory pain relief [22]. Furthermore, research indicates that hydromorphone hydrochloride injection is effective and exhibits lower toxicity in treating chronic pain, significantly improving sleep quality in patients [23]. Therefore, as an effective semi-synthetic opioid analgesic, hydromorphone hydrochloride can manage chronic pain with minimal adverse effects, facilitating safer outpatient care and greatly enhancing patients’ quality of life.

### Neuropathic pain

Neuropathic pain is defined as pain resulting from damage or disease in the somatosensory nervous system, including both peripheral and central neuropathic pain. Peripheral neuropathic pain commonly includes conditions such as postherpetic neuralgia, trigeminal neuralgia, and diabetic neuropathy, whereas central neuropathic pain is most frequently associated with post-stroke neuralgia. Patients often describe this pain as a tingling sensation, burning, or increased sensitivity to pain or temperature [18]. In clinical settings, anxiety and depression are prevalent in individuals suffering from postherpetic neuralgia. The intrathecal administration of low-dose hydromorphone has demonstrated effectiveness in alleviating severe, intractable craniofacial pain associated with postherpetic neuralgia [24], while also reducing the emotional, cognitive, and behavioral impacts of chronic pain, thereby improving overall quality of life, with no significant side effects reported after drug cessation [25]. Acute post-infectious polyneuropathic pain from Guillain-Barré syndrome can also be effectively treated with hydromorphone in combination with other medications [26] Furthermore, patients with central neuropathic pain due to radiation necrosis from stereotactic radiosurgery, particularly those with pontine metastases from lung cancer, have been effectively managed with a daily regimen of 25 mg methadone and 8 mg hydromorphone [27]. This substantial evidence reinforces the safety and efficacy of hydromorphone in neuropathic pain management, offering critical insights for future treatment approaches.

### Nociceptive pain

Nociceptive pain, resulting from tissue injury, is typically associated with damage to muscles, bones, skin, or visceral organs. Clinically, it is often observed in cases such as infectious inflammatory pain and osteoarthritis-related joint pain. The pain is generally described as sharp, throbbing, or pulsating [18]. Hydromorphone has been demonstrated to facilitate the translocation of PKC-positive neurons from the membrane to the cytoplasm in the L6 segment of the spinal cord, effectively alleviating stimuli caused by colorectal distension. Furthermore, it mitigates visceral pain by inhibiting the expression of various mRNAs and proteins within the spinal MAPK/ERK pathway [28]. In a patient with a long-standing history of gout complicated by gouty nephropathy, chronic kidney failure, and chronic gastritis with gastric ulcers, titration of hydromorphone *via* PCA resulted in rapid pain relief with minimal adverse effects [29]. Research indicates that both nonsteroidal anti-inflammatory drugs (NSAIDs) and hydromorphone provide similar analgesic effects in patients with knee osteoarthritis. These findings may assist clinicians and patients in discussing the potential advantages of alternative analgesics [30]. Although the research on hydromorphone in nociceptive pain remains limited, current data suggest its potential utility in this domain. Further clinical trials with larger samples are necessary to validate these findings.

## Adverse effects of hydromorphone

Hydromorphone may cause adverse effects through β-blocker-mediated signaling pathways [31]. Common side effects include nausea, vomiting, constipation, dizziness, urinary retention, and pruritus. These effects are primarily due to hydromorphone’s action on μ-opioid receptors, which are responsible for producing euphoria, constipation, nausea, vomiting, respiratory depression, drowsiness, and dependence [32]. In patients with renal impairment, hydromorphone’s neurotoxic effects, such as tremors and agitation, are more likely due to the accumulation of its metabolite H3G. For example, a patient with acute kidney injury and chronic kidney disease exhibited a series of neurotoxic reactions following intravenous hydromorphone administration, likely related to H3G buildup [33]. Cardiac effects have also been observed. Patients who received hydromorphone experienced bradycardia with prolonged sinus pauses up to 7.1 s, raising the possibility that hydromorphone may be the cause of vagal hyperexcitability because of its effect on cardiac sinus node delta receptors [34]. Although many patients do not experience severe toxicity, localized discomfort has been noted with subcutaneous injections of hydromorphone at high doses [3]. Despite these risks, the Centre for Oncology Research at Erasmus University Rotterdam in the Netherlands discovered that continuous intravenous hydromorphone helped treat severe, intractable cancer pain that had not responded to conventional opioids, with a comparable decrease in side effects [35]. Additionally, 10 cancer patients were selected as study subjects and given hydromorphone analgesia to explore the genetic variants associated with respiratory depression. Five single nucleotide polymorphisms were found to be associated with respiratory depression after hydro-morphone analgesia, which could provide some guidance for follow-up studies and clinical treatment, but further studies are needed to confirm its clinical significance [36].

## Conclusion and future perspectives

In conclusion, the global cancer patient population continues to rise, and with it, challenges in effective pain management become increasingly critical. In addition to difficulties in swallowing oral medications and pain in the later stages of life, most cancer patients experience persistent issues such as breakthrough pain, bone metastases, and other causes of pain. They also typically lack access to appropriate analgesics [37]. The current standard clinical analgesic solutions have large intravascular drug concentration fluctuations that make it difficult to achieve stable and satisfactory analgesic effects. Similarly, repeated subcutaneous or intravenous injections are short-lived and ineffective in relieving patients’ pain [38]. There is still a long way to go in cancer treatment around the world. Cancer patients must select a safe, effective, timely, and personalized analgesic technique since cancer pain has a significant negative impact on the quality of life for both patients and their families. Analgesic pump therapy has grown in popularity among patients, families, and medical professionals with advancements in pain medicine. Parenteral administration is the best option since advanced cancer patients frequently have digestive dysfunction in addition to their increased cancer incidence. These factors make cancer pain management a worry for both the medical community and the general public. According to the study, hydromorphone total dose PCA applied to home analgesia for advanced cancer pain is straightforward and easy to administer, family members find it easy to manage, analgesic safety is high, pain relief is effective, and nausea, vomiting, and constipation are significantly reduced, greatly improving the overall quality of life [38]. There is agreement, according to a significant number of studies, that intravenous hydromorphone should be used to treat advanced cancer pain. However, other studies have found that subcutaneous injection is secure and efficient, and because of its mobility, ease of use, and convenience, it is even regarded as the first option. Different modes of administration can be chosen to meet various analgesic needs, but there aren’t enough relevant studies to compare the efficacy of various modes of administration. As a result, large sample sizes and multicenter clinical trials are needed to confirm the efficacy and safety of various routes of administration.

In summary, the opioid receptor agonist hydromorphone demonstrates significant clinical efficacy due to its potent analgesic effects and excellent compatibility with medications of the same ph. In addition to treating pain in cancer patients, hydromorphone can also be used to alleviate pain in non-cancer patients. Hydromorphone has been used to treat various ailments, according to an increasing amount of domestic and international literature, although the amount of study on the drug is still rather modest and mostly focuses on comparing it to morphine. The role of hydromorphone may even become more important in the future with the development of hydromorphone in non-cancer pain.

## Figures and Tables

**Fig. 1 f1-pr74_41:**
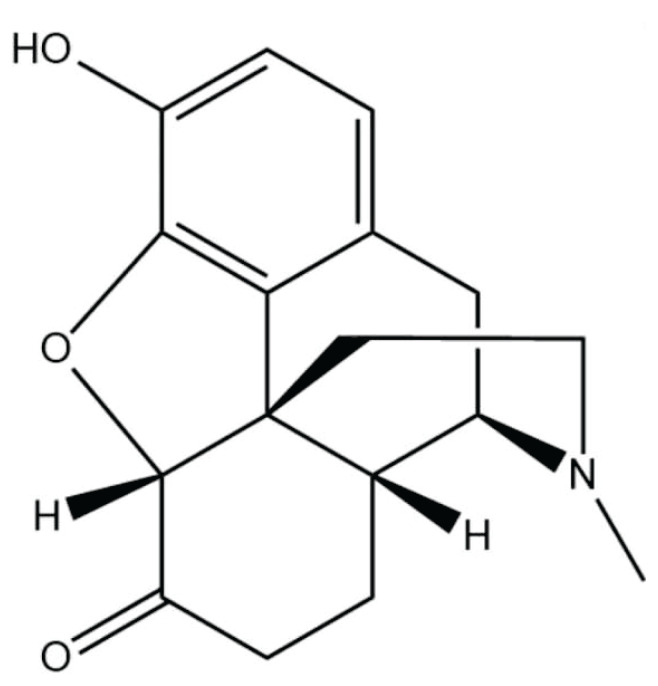
Chemical structure diagram of hydromorphone.
